# Teacher self-efficacy and thriving at work among junior high school physical education teachers: the mediating role of basic psychological need satisfaction

**DOI:** 10.3389/fpsyg.2026.1846810

**Published:** 2026-05-13

**Authors:** Hongfei Li, Renchao Lv, Chaohu He, Yuhong Wang

**Affiliations:** School of Physical Education, Kunming University, Kunming, China

**Keywords:** basic psychological need satisfaction, junior high school physical education teachers, Self-Determination Theory, teacher self-efficacy, thriving at work

## Abstract

**Introduction:**

Against the backdrop of ongoing school physical education reform and the Physical Education Entrance Examination in China, junior high school physical education teachers are facing increasing instructional and training demands, making their positive occupational functioning an important issue. This study examined the relationship between teacher self-efficacy and thriving at work among junior high school physical education teachers in China and tested the mediating role of basic psychological need satisfaction.

**Methods:**

A questionnaire survey was conducted among junior high school physical education teachers, and 609 valid responses were obtained. The instruments included the Physical Education Teachers’ Teaching Efficacy Scale, the Basic Psychological Need Satisfaction Scale, and the Thriving at Work Scale. Data were analyzed using reliability and validity tests, a common method bias test, descriptive statistics, correlation analysis, and PROCESS mediation analysis.

**Results:**

Teacher self-efficacy was positively associated with thriving at work and basic psychological need satisfaction. Basic psychological need satisfaction was also positively associated with thriving at work. Mediation analysis showed that basic psychological need satisfaction partially mediated the relationship between teacher self-efficacy and thriving at work.

**Discussion:**

These findings indicate that higher teacher self-efficacy not only directly promotes thriving at work among junior high school physical education teachers, but also indirectly enhances vitality and learning through increased basic psychological need satisfaction. This study provides empirical evidence for understanding the formation mechanism of positive occupational functioning among junior high school physical education teachers and offers practical implications for promoting their professional development and psychological support in the context of school physical education reform.

## 1 Introduction

In recent years, school physical education in China has entered a period of strong policy support. The Physical Education and Health Curriculum Standards for Compulsory Education, issued by the Ministry of Education in 2022, specified that physical education and health should account for 10–11% of total class hours, thereby reinforcing the position of physical education as a core subject within compulsory education. In 2025, the Ministry of Education further issued the Notice on Several Measures to Strengthen the Construction of Physical Education Teachers in Primary and Secondary Schools in the New Era, which required junior high schools to employ full-time physical education teachers at a class-teacher ratio of no more than 6:1. The policy also stipulated that responsibilities such as recess activities, physical fitness monitoring, after-school training, extracurricular activities, and coaching for competitions should be included in teachers’ workload, with the aim of ensuring parity with teachers of other subjects in professional evaluation, welfare, and recognition. These policy initiatives indicate a steady elevation in the status of physical education and a growing recognition of the professional value of physical education teachers.

However, the implementation of these policy goals has not been straightforward. As physical education class hours have increased and school time allocated to physical activity has been institutionally protected, structural tensions in teacher staffing have become increasingly salient. Public discussions in China have pointed out that curricular expansion has often been accompanied by heavier pressure on physical education teachers. Survey evidence from the Beijing Yao Foundation suggests that many physical education teachers work under moderate-to-high teaching intensity, with some teaching more than 16 classes per week while also undertaking after-school services, specialized training, physical fitness monitoring, and event organization. If sustained over time, this imbalance between rising task demands and insufficient staffing may not only reduce the attractiveness of the profession but also hinder the transition of school physical education from ensuring instructional time to improving educational quality.

Within this broader policy and occupational context, junior high school physical education teachers warrant particular attention. Junior high school is not only a critical stage for students’ physical and psychological development but also a period in which pressure associated with the Physical Education Entrance Examination becomes especially pronounced. In response to teacher shortages, some schools have adopted arrangements such as shared teaching and combined classes, with several classes or even an entire grade sometimes attending physical education simultaneously. Such arrangements intensify the demands placed on teachers in instructional organization, classroom management, safety control, and student engagement. Under these conditions, a central psychological question emerges: how can junior high school physical education teachers maintain vitality and continue to grow at work rather than merely cope with increasing demands?

This question is situated within a broader international concern regarding teachers’ positive occupational functioning. Teacher well-being has received increasing research attention because it is closely related to teacher retention, teacher–student relationships, and student outcomes (Dreer, 2023). A recent meta-analysis also showed that teacher well-being is embedded in a wider nomological network involving personal resources, occupational commitment, burnout, and work engagement (Zhou et al., 2024). From a job demands–resources perspective, teacher well-being is influenced by both job demands and job resources, indicating that teachers’ positive functioning should be understood in relation to the resources available to them as well as the demands they face (Li et al., 2025). These findings suggest that the psychological functioning of teachers cannot be adequately understood by focusing only on stress or burnout. It is also necessary to examine how teachers sustain energy, learning, and adaptive development under demanding work conditions.

Thriving at work provides a useful construct for addressing this issue. Spreitzerdefined thriving at work as a positive psychological state in which individuals experience both vitality and learning at work (Spreitzer et al., 2005). Vitality reflects a sense of energy and aliveness, whereas learning refers to the perception that one is continuously acquiring and applying new knowledge and skills. Porath et al. (2011) further demonstrated, through scale development and construct validation, that thriving at work consists of these two dimensions and predicts important outcomes such as proactive career development, health, burnout, and job performance(Porath et al., 2011). Compared with job satisfaction, which primarily reflects an attitudinal evaluation, and burnout, which focuses on resource depletion and impaired functioning, thriving at work more directly captures the dynamic and developmental qualities of positive occupational functioning. A meta-analysis by Kleine et al. (2019) further showed that thriving at work is associated with positive individual characteristics, supportive relational features, and important work outcomes, and that it provides incremental explanatory value beyond related constructs such as positive affect and work engagement (Kleine et al., 2019). Therefore, examining thriving at work among junior high school physical education teachers can help clarify how this group maintains vitality and learning under high work demands.

Teacher self-efficacy is a key psychological resource that may contribute to thriving at work. Rooted in Social Cognitive Theory, teacher self-efficacy refers to teachers’ beliefs about their capability to organize and implement instruction, manage classroom challenges, and promote student learning. Zee and Koomen (2016) synthesized 40 years of research and showed that teacher self-efficacy is positively associated with classroom processes, student academic adjustment, and teacher well-being. More recent evidence also indicates that teacher self-efficacy is important for a successful and healthy teaching career; a systematic review and meta-analysis of intervention studies found that teacher self-efficacy can be promoted through targeted interventions (Täschner et al., 2024). In addition, Skaalvik and Skaalvik (2014) found that teacher self-efficacy and perceived autonomy independently predicted teacher engagement, job satisfaction, and emotional exhaustion. These findings suggest that teacher self-efficacy is not merely a belief about instructional competence but also an important psychological resource related to teachers’ motivation, emotional functioning, and occupational adaptation.

The relevance of teacher self-efficacy may be particularly strong in physical education. Unlike many subjects that rely primarily on classroom-based instruction, physical education requires teachers to respond immediately to dynamic teaching situations, organize physical activities, demonstrate motor skills, regulate student behavior, manage safety risks, and maintain student participation. For junior high school physical education teachers, especially those working under the pressure of the Physical Education Entrance Examination, confidence in one’s ability to manage these demands may help sustain a sense of control, effort investment, and willingness to improve. Research on physical education teachers has shown that self-efficacy positively influences work engagement and creative teaching, suggesting that teachers with stronger efficacy beliefs are more likely to display active involvement and constructive instructional behaviors (Xiong et al., 2020). Evidence from Chinese teacher samples has also shown that teacher efficacy is significantly associated with work engagement (Minghui et al., 2018). Although few studies have directly examined the relationship between teacher self-efficacy and thriving at work, existing findings suggest a clear theoretical pathway: teachers with stronger self-efficacy are more likely to approach teaching challenges proactively, remain energetic, and continue learning from their work. Accordingly, this study proposes the following hypothesis:

*H1*: Teacher self-efficacy is positively associated with thriving at work.

Although teacher self-efficacy may directly contribute to thriving at work, this relationship is unlikely to operate only through a direct pathway. Basic psychological need satisfaction may represent an important psychological mechanism linking the two constructs. According to Self-Determination Theory, individuals have three basic psychological needs: autonomy, competence, and relatedness. Autonomy refers to the experience of volition and self-endorsement in one’s actions; competence refers to the feeling of being effective in dealing with environmental challenges; and relatedness refers to the experience of connection, care, and belonging. Satisfaction of these needs is considered essential for psychological growth, internalization, and well-being (Ryan and Deci, 2000). In work settings, den Van Broeck et al. (2016) showed that autonomy, competence, and relatedness satisfaction are consistently associated with psychological growth, internalization, and indicators of well-being, and that each need has unique predictive value.

In teaching contexts, basic psychological need satisfaction has also been shown to play an important role in teachers’ psychological functioning. Collie et al. (2016) found that perceived autonomy support predicted teachers’ basic psychological need satisfaction, which in turn predicted well-being, motivation, job satisfaction, and organizational commitment. Doménech-Betoret et al. (2015) found that need satisfaction was positively associated with work engagement and negatively associated with burnout among secondary school teachers, and that it mediated the relationships between teacher support resources and occupational outcomes. Jin et al. (2022) similarly showed that autonomy, competence, and relatedness satisfaction mediated the relationship between strengths use and teachers’ work engagement in a Chinese teacher sample. These studies indicate that need satisfaction is not a peripheral condition but a central mechanism through which personal and contextual resources are translated into positive occupational functioning.

Teacher self-efficacy is likely to be positively associated with basic psychological need satisfaction. Teachers with higher self-efficacy are more likely to believe that they can handle instructional challenges, solve classroom problems, and promote student progress. These competence-related beliefs may directly enhance competence satisfaction. In addition, teachers who believe that they can perform teaching tasks effectively may be more willing to act on their professional judgment, adjust instructional strategies, and take initiative in organizing teaching activities, thereby strengthening autonomy satisfaction. Higher self-efficacy may also facilitate more positive interactions with students, colleagues, and school administrators, which can enhance relatedness satisfaction. Kassis et al. (2019) provided relevant evidence in teacher education, showing that perceived self-efficacy was associated with higher levels of autonomy and competence satisfaction. Applied to junior high school physical education teachers, this suggests that teacher self-efficacy may help teachers experience their work as more manageable, self-directed, and socially connected. Therefore, this study proposes:

*H2*: Teacher self-efficacy is positively associated with basic psychological need satisfaction.

Basic psychological need satisfaction is also expected to be positively associated with thriving at work. Thriving at work consists of vitality and learning, and these two components correspond closely to the energy and growth aspects of positive psychological functioning. When teachers experience autonomy, they are more likely to perceive their work as self-endorsed rather than merely externally imposed. When they experience competence, they are more likely to feel capable of dealing with instructional challenges and improving their professional practice. When they experience relatedness, they are more likely to feel supported and connected in their school environment. These experiences can create the motivational and emotional conditions necessary for vitality and learning at work. Prior research has shown that need satisfaction supports teachers’ engagement, enjoyment, happiness, and adaptive work-related perceptions (Collie et al., 2016; Doménech-Betoret et al., 2015). Accordingly, this study proposes:

*H3*: Basic psychological need satisfaction is positively associated with thriving at work.

Taken together, Social Cognitive Theory and Self-Determination Theory provide a complementary theoretical basis for the present study. Social Cognitive Theory explains why teacher self-efficacy can function as a personal psychological resource: teachers who believe they can manage instructional demands are more likely to persist, invest effort, and respond adaptively to challenges. Self-Determination Theory explains how this personal resource may be translated into positive occupational functioning: efficacy beliefs may strengthen teachers’ experiences of competence, autonomy, and relatedness, which in turn support vitality and learning at work. Thus, basic psychological need satisfaction may serve as a mediating mechanism linking teacher self-efficacy to thriving at work. This mediation logic is especially relevant in the context of junior high school physical education, where teachers face increasing instructional, training, safety, and examination-related demands. Therefore, this study proposes:

*H4*: Basic psychological need satisfaction mediates the relationship between teacher self-efficacy and thriving at work.

The present study makes three contributions to the literature. First, it extends research on teacher self-efficacy by linking it to thriving at work, a positive occupational state that emphasizes both vitality and learning. Second, it clarifies the psychological mechanism underlying this relationship by examining the mediating role of basic psychological need satisfaction. Third, it provides context-specific evidence from junior high school physical education teachers in China, a group facing increasing policy expectations, workload demands, and examination-related pressure. By doing so, this study contributes to a more nuanced understanding of how teachers maintain positive occupational functioning under demanding educational conditions (Table 1).

**Table 1:** Hypotheses.

Serial number	Hypotheses
H1	Teachers’ self-efficacy is significantly positively correlated with their sense of work enthusiasm.
H2	Teachers’ self-efficacy is significantly positively correlated with the satisfaction of basic psychological needs.
H3	The satisfaction of basic psychological needs is significantly positively correlated with a sense of work enthusiasm.
H4	The satisfaction of basic psychological needs plays a mediating role between teachers’ self-efficacy and their sense of work enthusiasm.

## 2 Materials and methods

### 2.1 Participants, procedure, and sample size justification

This study targeted junior high school physical education teachers in Yunnan Province, China. A combination of convenience sampling and snowball sampling was used, and questionnaires were distributed through both online and offline channels. Participants were eligible if they were currently employed as physical education teachers in junior high schools in Yunnan Province and had at least one semester of teaching experience at the junior high school level.

An *a priori* power analysis was conducted using G*Power 3.1.9.7 to determine the minimum sample size required for the mediation-related regression analysis. The analysis was based on an F test for linear multiple regression, with a fixed model and R^2^ deviation from zero. The input parameters were set as follows: a medium effect size (f^2^ = 0.15), an alpha level of 0.05, statistical power of 0.95, and two predictors. The results indicated that the minimum required sample size was 107, with an actual power of 0.952. The main study retained 609 valid responses, which substantially exceeded the minimum sample size requirement. Therefore, the sample size was considered sufficient for the planned statistical analyses.

### 2.2 Pilot study

Before the main survey, a pilot study was conducted to assess the preliminary quality and applicability of the questionnaire among junior high school physical education teachers. The pilot study served four purposes. First, it was used to examine the preliminary reliability and validity of the three scales in the target population. Second, it helped assess whether the wording of the questionnaire items was clear, understandable, and free from ambiguity. Third, it allowed the researchers to estimate the time required to complete the questionnaire and to evaluate whether the overall length of the instrument was appropriate. Fourth, the pilot data provided preliminary evidence regarding whether the dimensional structure of the scales was consistent with theoretical expectations, thereby informing the subsequent measurement evaluation in the main study.

### 2.3 Measures

#### 2.3.1 Teacher self-efficacy

Teacher self-efficacy was measured using the Physical Education Teachers’ Teaching Efficacy Scale developed by Ma (2005) for Chinese physical education teachers. The scale consists of 20 items across four dimensions: classroom management, clarity of instructional presentation, application of teaching strategies and techniques, and teacher–student interaction, with five items in each dimension. Responses were rated on a 6-point Likert scale ranging from 1 (“completely unable to do so”) to 6 (“completely able to do so”). Some items were reverse scored, and higher scores indicated higher levels of teacher self-efficacy among physical education teachers.

Following the exploratory factor analysis in the current study, item A9 was removed because it did not meet the item retention criteria. Therefore, 19 items were retained for the final analysis of teacher self-efficacy.

#### 2.3.2 Basic psychological need satisfaction

Basic psychological need satisfaction was measured using a Chinese version of the Work-related Basic Need Satisfaction Scale (W-BNS) based on the instrument developed by den Van Broeck et al. (2010). Grounded in Self-Determination Theory, the scale assesses three dimensions of basic psychological need satisfaction: autonomy, competence, and relatedness. In the present study, the scale comprised 18 items, with six items for each dimension. Responses were rated on a 5-point Likert scale ranging from 1 (“strongly disagree”) to 5 (“strongly agree”). Some items were reverse scored, and higher scores indicated higher levels of basic psychological need satisfaction at work.

#### 2.3.3 Thriving at work

Thriving at work was measured using a Chinese version of the Thriving at Work Scale developed by Porath et al. (2011). The scale contains 10 items across two dimensions, vitality and learning, with five items in each dimension. Responses were rated on a 7-point Likert scale ranging from 1 (“strongly inconsistent”) to 7 (“strongly consistent”). Some items were reverse scored, and higher scores indicated higher levels of thriving at work.

### 2.4 Data analysis

Data were first screened and cleaned before statistical analysis. The data-cleaning procedures included checking questionnaire completeness, standardizing variable coding, verifying item entry accuracy, and recoding reverse-scored items to ensure that all items were scored in the same direction. After data screening, invalid questionnaires were excluded from subsequent analyses.

An *a priori* power analysis was conducted using G*Power 3.1.9.7 to determine the minimum sample size required for the planned regression-based mediation analysis. The analysis was based on an F test for linear multiple regression with a fixed model and R^2^ deviation from zero. The input parameters were set as follows: medium effect size (f^2^ = 0.15), *α* = 0.05, power = 0.95, and two predictors. The results indicated that the minimum required sample size was 107. The final valid sample size of the main study was 609, which exceeded this requirement.

All statistical analyses were conducted using SPSS, AMOS, and the PROCESS macro. First, reliability analyses were performed using Cronbach’s alpha coefficients. Second, exploratory factor analysis (EFA) was conducted to examine the underlying factor structure of the measurement items and to identify items that did not meet the predefined retention criteria. Items were retained when they showed adequate primary factor loadings and did not present problematic cross-loadings. Third, confirmatory factor analysis (CFA) was conducted to further evaluate the measurement model. Model fit was assessed using commonly reported fit indices, including χ^2^/df, CFI, TLI, RMSEA, and SRMR.

Construct reliability and validity were further evaluated using composite reliability (CR), average variance extracted (AVE), and discriminant validity evidence. CR was used to assess construct reliability, and AVE was used to evaluate convergent validity. Discriminant validity was examined by comparing the square root of AVE with the inter-construct correlations. Discriminant validity was considered acceptable when the square root of AVE for each construct was greater than its correlations with other constructs.

Given that the data were collected using self-report questionnaires at a single time point, Harman’s single-factor test was conducted to assess potential common method bias. Descriptive statistics and Pearson correlation coefficients were then calculated to examine the preliminary relationships among teacher self-efficacy, basic psychological need satisfaction, and thriving at work. Correlations between demographic variables and the core study variables were also examined to determine whether demographic variables should be included as controls in the mediation analysis.

Finally, the mediating effect of basic psychological need satisfaction in the relationship between teacher self-efficacy and thriving at work was tested using PROCESS Model 4. Teacher self-efficacy was specified as the independent variable, basic psychological need satisfaction as the mediator, and thriving at work as the dependent variable. Indirect effects were tested using a bias-corrected bootstrap procedure with 5,000 resamples and 95% confidence intervals. An indirect effect was considered significant when the confidence interval did not include zero. In addition to statistical significance, effect sizes were reported to evaluate the practical magnitude of the findings, including R^2^, Cohen’s f^2^, and the proportion of the total effect mediated. All statistical tests were two-tailed, and the significance level was set at *p* < 0.05.

## 3 Results

### 3.1 Screening of invalid questionnaires

Before the main analyses, the pilot-study and main-study data were screened and cleaned. The data-cleaning procedures included checking questionnaire completeness, standardizing variable coding, verifying item entry accuracy, and recoding reverse-scored items to ensure that all items were scored in the same direction. Questionnaires that could not be used for subsequent statistical analyses were excluded.

In the pilot study, 110 questionnaires were distributed, and 102 valid responses were retained, yielding an effective response rate of 92.73%. In the main study, 720 questionnaires were distributed, and 609 valid responses were retained, yielding an effective response rate of 84.58%. The cleaned main-study dataset was then used for reliability and validity analyses, common method bias testing, descriptive statistics, correlation analysis, and mediation analysis (Table 2).

**Table 2:** Questionnaire distribution and valid responses.

Survey stage	Questionnaires distributed	Valid responses retained	Effective response rate
Pilot study	110	102	92.73%
Main study	720	609	84.58%

### 3.2 Reliability and validity

#### 3.2.1 Pilot study reliability and validity

Before the main survey was conducted, a pilot study was carried out to assess the preliminary measurement quality of the questionnaire. The pilot-study sample consisted of 102 participants. Reliability analysis showed that the Cronbach’s alpha coefficients of the Teacher Self-Efficacy Scale, the Basic Psychological Need Satisfaction Scale, and the Thriving at Work Scale were 0.921, 0.923, and 0.919, respectively. All values exceeded 0.90, indicating high internal consistency for the three scales (Table 3).

**Table 3:** Reliability analysis of the pilot study.

Variable name	Number of items	Sample size	Cronbach’s *α* coefficient
Teacher self-efficacy	20	102	0.921
Basic psychological need satisfaction	18	102	0.923
Thriving at work	10	102	0.919

To further examine whether the scales were suitable for factor analysis, KMO and Bartlett’s tests of sphericity were conducted separately for the three scales. The results showed that the KMO values for the Teacher Self-Efficacy Scale, the Basic Psychological Need Satisfaction Scale, and the Thriving at Work Scale were 0.833, 0.914, and 0.896, respectively, all of which exceeded 0.80. Bartlett’s tests of sphericity were significant for all three scales: Teacher Self-Efficacy Scale, χ^2^ = 1013.198, df = 190, *p* < 0.001; Basic Psychological Need Satisfaction Scale, χ^2^ = 1197.966, df = 153, p < 0.001; and Thriving at Work Scale, χ^2^ = 591.562, df = 45, *p* < 0.001. These findings indicate that the items within each scale were sufficiently intercorrelated, the data were appropriate for subsequent factor analysis, and the questionnaire demonstrated acceptable preliminary structural validity (Table 4).

**Table 4:** Validity analysis of the pilot study.

Test	Statistic	Teacher self-efficacy	Basic psychological need satisfaction	Thriving at work
KMO		0.833	0.914	0.896
Bartlett sphericity test	Approximate chi-square	1013.198	1197.966	591.562
	*df*	190	153	45

Overall, the pilot-study results suggest that the Teacher Self-Efficacy Scale, the Basic Psychological Need Satisfaction Scale, and the Thriving at Work Scale demonstrated satisfactory preliminary reliability and validity and were therefore suitable for use in the main study.

#### 3.2.2 Exploratory factor analysis

To strengthen the evaluation of the measurement structure, the main-study sample was randomly divided into two independent subsamples for cross-validation. Exploratory factor analysis (EFA) was conducted using the first subsample (*n* = 319), whereas confirmatory factor analysis (CFA) was subsequently conducted using the second subsample (*n* = 290). This split-sample strategy was adopted to avoid evaluating and confirming the factor structure in the same dataset, thereby reducing capitalization on chance and providing a more rigorous assessment of the measurement model (Table 5).

**Table 5:** Split-sample strategy for measurement validation.

Sample	*n*	Purpose	Analysis
Subsample 1	319	Factor structure exploration and item purification	EFA
Subsample 2	290	Independent validation of the measurement model	CFA
Total sample	609	Main-study sample	Split-sample cross-validation

The main-study sample was randomly divided into two independent subsamples. EFA was conducted in Subsample 1, and CFA was conducted in Subsample 2 to provide cross-validation of the measurement model.

EFA was performed using principal axis factoring with Kaiser-normalized oblimin rotation, because the core constructs were theoretically expected to be correlated. In the first round of EFA, all 48 items were included. The results showed that the data were suitable for factor analysis, with a KMO value of 0.926 and a significant Bartlett’s test of sphericity, χ^2^ = 8949.696, df = 1,128, *p* < 0.001. A three-factor structure was extracted, corresponding to teacher self-efficacy, basic psychological need satisfaction, and thriving at work. However, item A9 showed a relatively low communality after extraction (0.273) and its primary factor loading in the pattern matrix was 0.476, which did not meet the predefined item retention criterion of a primary loading greater than 0.50. Therefore, item A9 was removed from the subsequent analysis (Table 6).

**Table 6:** Summary of exploratory factor analysis before and after item deletion.

EFA round	Items included	KMO	Bartlett’s χ^2^	df	*p*	Number of factors	Cumulative variance explained	Decision
Initial EFA	48	0.926	8949.696	1,128	*p* < 0.001	3	46.680%	Item A9 removed
Revised EFA	47	0.928	8783.015	1,081	*p* < 0.001	3	47.094%	Retained

Principal axis factoring with Kaiser-normalized oblimin rotation was used. Item A9 was removed because its communality after extraction was 0.273 and its primary loading in the pattern matrix was below 0.50.

After deleting item A9, the EFA was rerun with the remaining 47 items. The revised analysis showed a slightly improved KMO value of 0.928, and Bartlett’s test of sphericity remained significant, χ^2^ = 8783.015, df = 1,081, *p* < 0.001. The three extracted factors explained 47.094% of the total variance. The pattern matrix showed a clear three-factor structure: the 18 basic psychological need satisfaction items loaded on the first factor, the 19 retained teacher self-efficacy items loaded on the second factor, and the 10 thriving at work items loaded on the third factor. The primary factor loadings ranged from 0.581 to 0.780 for basic psychological need satisfaction, from 0.571 to 0.721 for teacher self-efficacy, and from 0.604 to 0.829 for thriving at work. No retained item showed problematic cross-loading in the pattern matrix. These results indicate that, after item purification, the retained items demonstrated a clear and theoretically consistent three-factor structure, providing a sound basis for the subsequent CFA (Table 7).

**Table 7:** Pattern matrix of the revised exploratory factor analysis.

Item	Factor 1: basic psychological need satisfaction	Factor 2: teacher self-efficacy	Factor 3: thriving at work
B13	0.780		
B16	0.777		
B15	0.747		
B4	−0.738		
B2	0.736		
B10	0.725		
B17	0.704		
B11	0.701		
B3	0.700		
B1	0.697		
B6	0.695		
B18	0.691		
B7	0.684		
B5	0.678		
B9	0.671		
B14	0.656		
B12	0.624		
B8	0.581		
A19		0.721	
A4		0.711	
A18		0.697	
A1		0.686	
A2		0.679	
A14		0.676	
A11		0.668	
A13		0.660	
A5		0.656	
A12		0.635	
A6		0.630	
A3		0.622	
A10		0.615	
A16		0.612	
A17		0.606	
A15		0.604	
A7		0.597	
A8		0.595	
A20		0.571	
C9			0.829
C10			0.761
C8			0.740
C3			0.682
C1			0.672
C5			0.646
C4			0.638
C7			0.631
C2			0.617
C6			0.604

Only primary factor loadings are shown for clarity. Extraction method: principal axis factoring. Rotation method: Kaiser-normalized oblimin rotation. Item A9 was deleted before the revised EFA.

#### 3.2.3 Confirmatory factor analysis

Confirmatory factor analysis (CFA) was conducted using the second independent subsample (*n* = 290) to validate the measurement model identified in the exploratory factor analysis. Because the retained measurement model contained 47 items, directly estimating all item-level parameters would have resulted in a highly complex model. Therefore, an item parceling strategy was adopted. Specifically, the retained items were aggregated into nine subdimension indicators corresponding to the theoretically defined dimensions of the three constructs: teacher self-efficacy, basic psychological need satisfaction, and thriving at work (Table 8).

**Table 8:** Fit indices of the confirmatory factor analysis model.

Model	χ^2^	df	p	χ^2^/df	CFI	TLI	RMSEA	SRMR
Three-factor measurement model	27.187	24	0.296	1.133	0.999	0.998	0.021	0.020

CFA was conducted using the second independent subsample (*n* = 290). Item parceling was applied, and nine subdimension indicators were used to represent the three latent constructs. CFI = comparative fit index; TLI = Tucker–Lewis index; RMSEA = root mean square error of approximation; SRMR = standardized root mean square residual.

The CFA results indicated that the three-factor measurement model showed good fit to the data: χ^2^ = 27.187, df = 24, *p* = 0.296, χ^2^/df = 1.133, CFI = 0.999, TLI = 0.998, RMSEA = 0.021, and SRMR = 0.020. These fit indices met commonly accepted criteria, suggesting that the three-factor measurement model was well supported in the independent validation subsample. Overall, the CFA results provided further evidence for the structural validity of the measurement model and supported the use of the retained measurement indicators in subsequent analyses.

#### 3.2.4 Construct reliability and validity

Construct reliability and validity were further examined using Cronbach’s alpha, composite reliability (CR), average variance extracted (AVE), and discriminant validity indicators. After item purification, the Teacher Self-Efficacy Scale retained 19 items, the Basic Psychological Need Satisfaction Scale retained 18 items, and the Thriving at Work Scale retained 10 items. The Cronbach’s alpha coefficients were 0.935 for teacher self-efficacy, 0.924 for basic psychological need satisfaction, and 0.916 for thriving at work, indicating good internal consistency for all three constructs.

The results also showed satisfactory composite reliability and convergent validity. The CR values were 0.929 for teacher self-efficacy, 0.940 for basic psychological need satisfaction, and 0.931 for thriving at work, all exceeding the commonly recommended threshold of 0.70. The AVE values were 0.767, 0.840, and 0.871, respectively, all exceeding 0.50. These results indicate that the three latent constructs demonstrated good construct reliability and convergent validity (Table 9).

**Table 9:** Construct reliability and convergent validity.

Construct	Indicator	Standardized loading	Cronbach’s α	AVE	CR
Teacher self-efficacy	Teacher–student interaction	0.903	0.935	0.767	0.929
	Instructional strategies	0.867			
	Clarity of instructional presentation	0.859			
	Classroom management	0.874			
Basic psychological need satisfaction	Relatedness	0.943	0.924	0.840	0.940
	Competence	0.947			
	Autonomy	0.856			
Thriving at work	Learning	0.929	0.916	0.871	0.931
	Vitality	0.938			

Teacher self-efficacy was measured using 19 retained items after item A9 was removed. AVE = average variance extracted; CR = composite reliability.

Discriminant validity was assessed by comparing the square root of AVE with the correlations among the latent constructs. The square roots of AVE were 0.876 for teacher self-efficacy, 0.917 for basic psychological need satisfaction, and 0.933 for thriving at work. These values were greater than the corresponding inter-construct correlations, which ranged from 0.309 to 0.629. Therefore, the three constructs showed acceptable discriminant validity (Table 10).

**Table 10:** Discriminant validity matrix.

Construct	F1	F2	F3	√AVE
F1 Teacher self-efficacy	0.767			0.876
F2 Basic psychological need satisfaction	0.309	0.840		0.917
F3 Thriving at work	0.458	0.629	0.871	0.933

Diagonal values are AVE values; lower-triangular values are inter-construct correlations; the last column presents the square root of AVE. Discriminant validity is supported when the square root of AVE is greater than the corresponding inter-construct correlations.

### 3.3 Common method bias test

Given that the data in this study were collected primarily through self-report questionnaires at a single time point, Harman’s single-factor test was conducted to assess potential common method bias. The results showed that the KMO value was 0.949, and Bartlett’s test of sphericity was significant, χ^2^ = 16904.929, df = 1,081, *p* < 0.001, indicating that the data were suitable for factor analysis. Under the unrotated solution, the first common factor had an eigenvalue of 15.053 and explained 32.027% of the total variance. Because the variance explained by the first common factor was below the commonly used threshold of 40%, common method bias was unlikely to pose a serious threat to the findings of this study (Table 11).

**Table 11:** Common method bias test.

Indicator	Value
Eigenvalue of the first factor before rotation	15.053
Variance explained by the first factor before rotation	32.027%
Cumulative variance explained before rotation	32.027%
KMO	0.949
Bartlett’s test of sphericity, χ^2^	16904.929
df	1,081
*p*	*p* < 0.001

Harman’s single-factor test was conducted using the unrotated factor solution. Common method bias was considered not serious because the first factor explained less than 40% of the total variance.

### 3.4 Descriptive statistics and correlations

#### 3.4.1 Descriptive statistics

Table 12 presents the descriptive statistics for the three core variables. Based on the mean scores across scale items, the mean values of thriving at work, basic psychological need satisfaction, and teacher self-efficacy were 4.325 (SD = 1.495), 3.483 (SD = 0.871), and 3.932 (SD = 1.179), respectively. The observed score ranges indicated that all three variables showed sufficient individual variation in the main-study sample. Among the three variables, thriving at work showed the highest mean score, whereas basic psychological need satisfaction showed the lowest mean score.

**Table 12:** Descriptive statistics of the core variables.

Variable	*N*	Minimum	Maximum	M	SD	Median
Thriving at work	609	1.500	6.600	4.325	1.495	4.600
Basic psychological need satisfaction	609	1.444	4.444	3.483	0.871	3.944
Teacher self-efficacy	609	1.474	5.737	3.932	1.179	3.737

Scores were calculated as mean scores across scale items. Teacher self-efficacy was calculated based on the 19 retained items after item A9 was removed.

Overall, the descriptive statistics indicated that the core variables had reasonable distributional characteristics, providing a basis for the subsequent correlation and mediation analyses.

#### 3.4.2 Correlations among the core variables

Pearson correlation analysis was conducted to examine the preliminary associations among teacher self-efficacy, basic psychological need satisfaction, and thriving at work. The results showed that teacher self-efficacy was significantly and positively correlated with thriving at work (r = 0.425, *p* < 0.001), indicating preliminary support for Hypothesis 1. Teacher self-efficacy was also significantly and positively correlated with basic psychological need satisfaction (r = 0.280, p < 0.001), providing preliminary support for Hypothesis 2. In addition, basic psychological need satisfaction was significantly and positively correlated with thriving at work (r = 0.593, p < 0.001), providing preliminary support for Hypothesis 3 (Table 13).

**Table 13:** Descriptive statistics and correlations among the core variables.

Variable	M	SD	1	2	3
1. Teacher self-efficacy	3.932	1.179	—		
2. Basic psychological need satisfaction	3.483	0.871	0.280**	—	
3. Thriving at work	4.325	1.495	0.425**	0.593**	—

*N* = 609. Scores were calculated as mean scores across scale items. Teacher self-efficacy was calculated based on the 19 retained items after item A9 was removed. **p < 0.01.

Overall, the significant positive correlations among the three core variables were consistent with the proposed theoretical model. These findings suggest that junior high school physical education teachers with higher levels of teacher self-efficacy tended to report higher levels of basic psychological need satisfaction and thriving at work, and that teachers with higher basic psychological need satisfaction tended to experience higher thriving at work. These correlation results provided an empirical basis for the subsequent mediation analysis.

#### 3.4.3 Correlations between demographic variables and the core variables

To determine whether demographic variables should be included as control variables in the subsequent mediation analysis, Pearson correlation analyses were conducted between demographic characteristics and the three core variables. The demographic variables included gender, age, teaching experience, educational attainment, professional title, school location, Grade 9 teaching assignment, homeroom teacher role, and weekly physical education teaching load (Table 14).

**Table 14:** Correlations between demographic variables and the core variables.

Core variable	Gender	Age	Teaching experience	Education	Professional title	School location	Grade 9 teaching assignment	Homeroom teacher role	Weekly PE teaching load
Basic psychological need satisfaction	−0.038	−0.025	−0.015	0.104**	−0.021	0.015	−0.032	0.009	0.066
Teacher self-efficacy	−0.039	0.068	0.001	0.009	0.007	−0.009	−0.005	0.001	0.012
Thriving at work	0.004	−0.025	0.062	0.051	0.015	0.053	−0.000	−0.018	0.017

*N* = 609. Values are Pearson correlation coefficients. **p < 0.01.

The results showed that most demographic variables were not significantly correlated with basic psychological need satisfaction, teacher self-efficacy, or thriving at work. Specifically, gender, age, teaching experience, professional title, school location, Grade 9 teaching assignment, homeroom teacher role, and weekly physical education teaching load were not significantly associated with any of the three core variables. Only educational attainment was significantly and positively correlated with basic psychological need satisfaction (r = 0.104, *p* = 0.010), whereas its correlations with teacher self-efficacy and thriving at work were not significant.

Given that the demographic variables did not show stable or systematic associations with the core study variables, they were not included as control variables in the mediation analysis. Although educational attainment was weakly associated with basic psychological need satisfaction, this isolated and small correlation was not considered sufficient to justify its inclusion as a control variable. This decision helped avoid unnecessary model complexity and reduced the risk of over control, thereby allowing the mediation model to focus more directly on the relationships among teacher self-efficacy, basic psychological need satisfaction, and thriving at work.

### 3.5 Mediation analysis

#### 3.5.1 Mediation model testing

To examine whether basic psychological need satisfaction mediated the relationship between teacher self-efficacy and thriving at work, mediation analysis was conducted using PROCESS Model 4. Teacher self-efficacy was entered as the independent variable, basic psychological need satisfaction as the mediator, and thriving at work as the dependent variable (Table 15).

**Table 15:** Regression results for the mediation model.

Outcome variable	Predictor	B	SE	t	*p*	β	R^2^	Adjusted R^2^	F
Thriving at work	Teacher self-efficacy	0.539	0.047	11.564	<0.001	0.425	0.181	0.179	133.717***
Basic psychological need satisfaction	Teacher self-efficacy	0.207	0.029	7.188	<0.001	0.280	0.078	0.077	51.671***
Thriving at work	Teacher self-efficacy	0.356	0.041	8.747	<0.001	0.281	0.424	0.422	222.918***
Thriving at work	Basic psychological need satisfaction	0.882	0.055	15.999	<0.001	0.514	0.424		

*N* = 609. The first row represents the total-effect model. The second row represents the mediator model. The third and fourth rows represent the final mediation model. ***p < 0.001.

The results showed that teacher self-efficacy significantly and positively predicted thriving at work in the total-effect model (B = 0.539, SE = 0.047, t = 11.564, *p* < 0.001). Teacher self-efficacy also significantly and positively predicted basic psychological need satisfaction (B = 0.207, SE = 0.029, t = 7.188, *p* < 0.001). When teacher self-efficacy and basic psychological need satisfaction were simultaneously entered into the model, basic psychological need satisfaction significantly predicted thriving at work (B = 0.882, SE = 0.055, t = 15.999, *p* < 0.001), and the direct effect of teacher self-efficacy on thriving at work remained significant (B = 0.356, SE = 0.041, t = 8.747, *p* < 0.001) (Table 16).

**Table 16:** Direct, indirect, and total effects.

Effect path	Symbol	Effect	SE	95% CI lower	95% CI upper	z/t	*p*	Conclusion
Teacher self-efficacy → Basic psychological need satisfaction → Thriving at work	a × b	0.182	0.029	0.131	0.242	6.393	<0.001	Partial mediation
Teacher self-efficacy → Basic psychological need satisfaction	a	0.207	0.029	0.150	0.264	7.188	<0.001	
Basic psychological need satisfaction → Thriving at work	b	0.882	0.055	0.773	0.990	15.999	<0.001	
Teacher self-efficacy → Thriving at work	c′	0.356	0.041	0.276	0.436	8.747	<0.001	
Teacher self-efficacy → Thriving at work	c	0.539	0.047	0.447	0.630	11.564	<0.001	

Bootstrap confidence intervals were based on 5,000 resamples. The indirect effect was significant because the 95% confidence interval did not include zero. c = total effect; c′ = direct effect; a × b = indirect effect.

**Figure 1:**
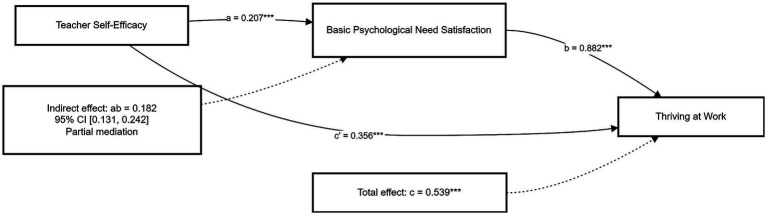
Mediation model. *p < 0.001.

The bootstrap results further indicated that the indirect effect of teacher self-efficacy on thriving at work through basic psychological need satisfaction was significant (indirect effect = 0.182, SE = 0.029, 95% CI [0.131, 0.242]). Because the confidence interval did not include zero, the mediating effect was significant. Since both the indirect effect and the direct effect remained significant, basic psychological need satisfaction partially mediated the relationship between teacher self-efficacy and thriving at work. Therefore, Hypothesis 4 was supported (Figure 1).

#### 3.5.2 Effect size of the mediation model

Effect size indicators were further examined to evaluate the practical magnitude of the mediation model. The total-effect model explained 18.1% of the variance in thriving at work (R^2^ = 0.181), corresponding to Cohen’s f^2^ = 0.221. The model predicting basic psychological need satisfaction explained 7.8% of the variance (R^2^ = 0.078), corresponding to Cohen’s f^2^ = 0.085. In the final mediation model, teacher self-efficacy and basic psychological need satisfaction jointly explained 42.4% of the variance in thriving at work (R^2^ = 0.424), corresponding to Cohen’s f^2^ = 0.736. This value indicates a large effect size for the final model (Table 17).

**Table 17:** Effect size estimates for the mediation model.

Model/Effect indicator	Value	Interpretation
R^2^ for total-effect model	0.181	18.1% variance explained
Cohen’s f^2^ for total-effect model	0.221	Medium-to-large effect
R^2^ for mediator model	0.078	7.8% variance explained
Cohen’s f^2^ for mediator model	0.085	Small-to-medium effect
R^2^ for final mediation model	0.424	42.4% variance explained
Cohen’s f^2^ for final mediation model	0.736	Large effect
Proportion mediated	33.874%	Partial mediation

Cohen’s f^2^ was calculated as R^2^/(1 − R^2^). The proportion mediated was calculated as the indirect effect divided by the total effect.

In addition, the indirect effect accounted for 33.874% of the total effect, indicating that approximately one-third of the association between teacher self-efficacy and thriving at work was transmitted through basic psychological need satisfaction. These findings suggest that the mediation effect was not only statistically significant but also meaningful in magnitude.

## 4 Discussion

### 4.1 Main findings

This study examined the relationships among teacher self-efficacy, basic psychological need satisfaction, and thriving at work among junior high school physical education teachers in China. The results showed that teacher self-efficacy was positively associated with thriving at work and basic psychological need satisfaction, and that basic psychological need satisfaction was positively associated with thriving at work. Further mediation analysis revealed that basic psychological need satisfaction partially mediated the relationship between teacher self-efficacy and thriving at work. These findings indicate that teacher self-efficacy not only directly contributes to thriving at work but also indirectly promotes it by enhancing teachers’ experiences of autonomy, competence, and relatedness.

These findings are consistent with the broader literature on teacher well-being and positive occupational functioning. Recent systematic and meta-analytic evidence has shown that teacher well-being is closely related to teacher retention, teacher–student relationships, student outcomes, occupational commitment, burnout, and work engagement (Dreer, 2023; Zhou et al., 2024). From this perspective, thriving at work can be understood as a specific form of positive occupational functioning that emphasizes both energy and development. The present study extends this line of research by focusing on junior high school physical education teachers, a group whose work is characterized by high instructional, organizational, safety-related, and examination-related demands. By identifying teacher self-efficacy and basic psychological need satisfaction as important psychological factors associated with thriving at work, this study provides empirical evidence for understanding how teachers maintain vitality and learning under demanding educational conditions.

### 4.2 Teacher self-efficacy and thriving at work

The present study found that teacher self-efficacy significantly and positively predicted thriving at work among junior high school physical education teachers. This finding suggests that teachers who have stronger beliefs in their ability to organize classes, implement instructional strategies, manage classroom challenges, and promote student participation are more likely to experience vitality and learning at work. This result supports Hypothesis 1 and indicates that teacher self-efficacy is not only a competence-related belief associated with instructional performance, but also a psychological resource that contributes to teachers’ positive occupational functioning.

This finding is consistent with previous research showing that teacher self-efficacy is closely related to adaptive motivational and emotional outcomes. Zee and Koomen (2016) found that teacher self-efficacy is positively associated with classroom processes, student academic adjustment, and teacher well-being. Skaalvik and Skaalvik (2014) further showed that teacher self-efficacy and perceived autonomy independently predicted teachers’ engagement, job satisfaction, and emotional exhaustion. More recent research has also linked teacher self-efficacy with reduced burnout, suggesting that teachers with stronger efficacy beliefs are better able to cope with occupational demands and maintain healthier professional functioning (Li, 2023). The present study extends these findings by showing that teacher self-efficacy is also associated with thriving at work, a construct that captures the simultaneous experience of vitality and learning rather than only satisfaction, engagement, or reduced burnout.

This relationship may be particularly meaningful in the context of physical education. Physical education teaching is highly dynamic and requires teachers to respond continuously to changing classroom and activity conditions. Compared with many classroom-based subjects, physical education places greater demands on real-time organization, behavioral regulation, motor skill demonstration, safety management, and student motivation. For junior high school physical education teachers, these demands are further intensified by the Physical Education Entrance Examination. Under such conditions, teachers with higher self-efficacy may be more likely to interpret work challenges as manageable rather than overwhelming. They may also be more willing to invest effort, adjust instructional strategies, and learn from teaching practice. These processes help explain why teacher self-efficacy is associated not only with work engagement and creative teaching among physical education teachers (Xiong et al., 2020), but also with the broader positive state of thriving at work.

### 4.3 The mediating role of basic psychological need satisfaction

The results showed that teacher self-efficacy significantly and positively predicted basic psychological need satisfaction, which in turn significantly and positively predicted thriving at work. In addition, basic psychological need satisfaction partially mediated the relationship between teacher self-efficacy and thriving at work. These findings support Hypotheses 2, 3, and 4. They suggest that teacher self-efficacy promotes thriving at work not only through a direct pathway, but also by strengthening teachers’ experiences of autonomy, competence, and relatedness in the workplace.

The positive association between teacher self-efficacy and basic psychological need satisfaction is theoretically meaningful. According to Self-Determination Theory, competence satisfaction refers to the experience of being effective in dealing with environmental demands, autonomy satisfaction refers to the experience of volition and self-endorsement, and relatedness satisfaction refers to the experience of connection and belonging. Teachers with higher self-efficacy are more likely to believe that they can manage teaching tasks, solve instructional problems, and support student progress. Such beliefs may directly enhance competence satisfaction. At the same time, self-efficacious teachers may feel more confident in making professional decisions and adapting teaching strategies, thereby enhancing autonomy satisfaction. They may also engage more positively with students, colleagues, and administrators, which can strengthen relatedness satisfaction. This interpretation is supported by Kassis et al. (2019), who found that perceived self-efficacy was associated with higher levels of autonomy and competence satisfaction in teacher education contexts.

The finding that basic psychological need satisfaction positively predicted thriving at work is also consistent with prior research on teachers’ psychological functioning. Collie et al. (2016) found that teachers’ need satisfaction predicted well-being, motivation, job satisfaction, and organizational commitment. Doménech-Betoret et al. (2015) similarly showed that need satisfaction was positively associated with work engagement and negatively associated with burnout among secondary school teachers. These findings suggest that basic psychological need satisfaction is a central mechanism through which teachers’ work environments and personal resources are translated into adaptive occupational outcomes. In the present study, this mechanism was extended to thriving at work. When junior high school physical education teachers feel autonomous, competent, and connected, they are more likely to maintain energy and derive learning from their work, even in demanding teaching contexts.

The partial mediation result deserves particular attention. It indicates that basic psychological need satisfaction explains part, but not all, of the relationship between teacher self-efficacy and thriving at work. This suggests that teacher self-efficacy may influence thriving through multiple pathways. For example, self-efficacious teachers may experience stronger professional identity, higher work engagement, more adaptive emotion regulation, or greater resilience, which may also contribute to their vitality and learning. The present findings therefore support basic psychological need satisfaction as an important mediating mechanism while also leaving room for future research to examine additional psychological and contextual mediators. This interpretation is consistent with the broader literature suggesting that teachers’ positive functioning is shaped by both personal resources and work-related resources.

### 4.4 Practical implications

The findings of this study have several practical implications for supporting junior high school physical education teachers. First, school administrators should regard the enhancement of teacher self-efficacy as an important entry point for promoting teachers’ positive occupational development. Teacher self-efficacy is not a fixed trait; rather, it can be strengthened through appropriate professional experiences and interventions. A recent systematic review and meta-analysis showed that interventions can significantly promote teacher self-efficacy, especially when they provide opportunities for practice, reflection, and mastery experiences (Täschner et al., 2024). Therefore, schools should provide physical education teachers with structured opportunities to accumulate successful teaching experiences through peer observation, instructional coaching, school-based professional development, targeted training, and constructive feedback.

Second, interventions should not focus only on improving individual teaching skills. They should also create work conditions that satisfy teachers’ basic psychological needs. For physical education teachers, autonomy support may involve allowing reasonable flexibility in lesson design, training arrangements, and instructional strategies. Competence support may involve providing clear teaching feedback, professional development in classroom safety and student motivation, and opportunities to improve teaching and training quality. Relatedness support may involve strengthening collaboration among physical education teachers, improving communication with school leaders, and increasing recognition of the professional value of physical education. Such support is particularly important under the Physical Education Entrance Examination system, where teachers face both instructional and performance-related pressures.

Third, school physical education management should move beyond a narrow focus on class-hour implementation, examination performance, or competition results. From a job demands–resources perspective, teacher well-being is shaped by the balance between demands and resources. Therefore, educational administrators should not simply increase expectations for physical education teachers without also improving the resources available to them. In practice, this means improving workload recognition, optimizing the allocation of teaching and training tasks, reducing inefficient administrative burdens, strengthening collegial collaboration, and establishing professional and psychological support mechanisms for physical education teachers. These measures may help transform policy pressure into sustainable professional growth and support the long-term occupational development of junior high school physical education teachers.

### 4.5 Limitations and future directions

Several limitations should be acknowledged. First, this study adopted a cross-sectional design, which prevents strict causal inferences regarding the relationships among teacher self-efficacy, basic psychological need satisfaction, and thriving at work. Although the mediation analysis was consistent with the proposed theoretical model, the results should not be interpreted as direct evidence of causal ordering. Future research should use longitudinal, time-lagged, or diary designs to examine the temporal dynamics of these variables and to clarify whether changes in teacher self-efficacy and need satisfaction predict subsequent changes in thriving at work.

Second, the study relied primarily on self-report questionnaire data. Although common method bias testing suggested that this issue was unlikely to seriously threaten the findings, same-source data may still involve social desirability bias, common method variance, or inflated subjective associations. Future studies could use multi-source and multi-method designs, such as combining teacher self-reports with peer evaluations, school administrator ratings, classroom observations, or qualitative interviews. Such approaches would provide a more comprehensive understanding of how teacher self-efficacy and need satisfaction operate in everyday physical education teaching.

Third, the sample consisted of junior high school physical education teachers in China. This focus enhances the contextual relevance of the study, but it also limits the generalizability of the findings. Junior high school physical education teachers work under distinctive pressures associated with the Physical Education Entrance Examination, safety management, student participation, and school physical education reform. Their experiences may differ from those of primary school physical education teachers, senior high school physical education teachers, or teachers of other subjects. Future research should therefore conduct cross-regional, cross-school-level, and cross-disciplinary comparisons to examine whether the present model is stable across different teacher groups and educational contexts.

Finally, although this study examined basic psychological need satisfaction as a mediator, the partial mediation effect suggests that other mechanisms may also explain the relationship between teacher self-efficacy and thriving at work. Future studies could examine additional mediators such as work engagement, professional identity, teacher resilience, emotion regulation, psychological capital, or perceived organizational support. Moreover, future research may integrate individual-level and organizational-level variables to better explain how personal resources and school environments jointly shape teachers’ positive occupational functioning. This would help develop a more comprehensive model of thriving at work among physical education teachers.

## 5 Conclusion

Teacher self-efficacy significantly and positively predicted thriving at work among junior high school physical education teachers, and basic psychological need satisfaction partially mediated this relationship. In other words, higher teacher self-efficacy not only directly promoted thriving at work, but also further enhanced vitality and learning by increasing teachers’ experiences of autonomy, competence, and relatedness at work. These findings provide new evidence for understanding the formation mechanism of positive occupational functioning among junior high school physical education teachers and offer practical implications for promoting their professional development and psychological support in the context of school physical education reform.

## Data Availability

The original contributions presented in the study are included in the article/Supplementary material, further inquiries can be directed to the corresponding authors.
